# Advances and future perspectives in the treatment and prognosis of type 1 diabetes mellitus

**DOI:** 10.3389/fcdhc.2025.1651061

**Published:** 2025-10-10

**Authors:** Yuqing Fu, Jun Zeng, Qian He

**Affiliations:** ^1^ Department of Endocrinology, The First College of Clinical Medical Science, China Three Gorges University & Yichang Central People’s Hospital, Yichang, China; ^2^ Yichang Key Laboratory of Endocrinology, Yichang, China; ^3^ Third-grade Pharmacological Laboratory on Traditional Chinese Medicine, State Administration of Traditional Chinese Medicine, China Three Gorges University, Yichang, China; ^4^ Department of Geriatric, The First College of Clinical Medical Science, China Three Gorges University & Yichang Central People’s Hospital, Yichang, China

**Keywords:** type 1 diabetes mellitus, immunotherapy, stem cell therapy, gene editing technology, drug therapy

## Abstract

Type 1 diabetes mellitus (T1DM) is an autoimmune disorder characterized by the destruction of pancreatic β-cells, necessitating lifelong exogenous insulin. This review synthesizes key advances that are shifting T1DM management from symptomatic control to disease modification and potential cure. We examine progress in novel insulin formulations and automated insulin delivery systems, alongside groundbreaking immunomodulatory therapies and gene-edited stem cell therapies that aim to restore native β-cell function and achieve insulin independence. The article also discusses the potential of phytomedicines and gut microbiota modulation. This review provides insights into the unique challenges of implementing these innovations within the Chinese healthcare context, highlighting the need for high-quality clinical research, personalized strategies, and improved healthcare accessibility to enhance long-term patient outcomes.

## Graphical Abstract

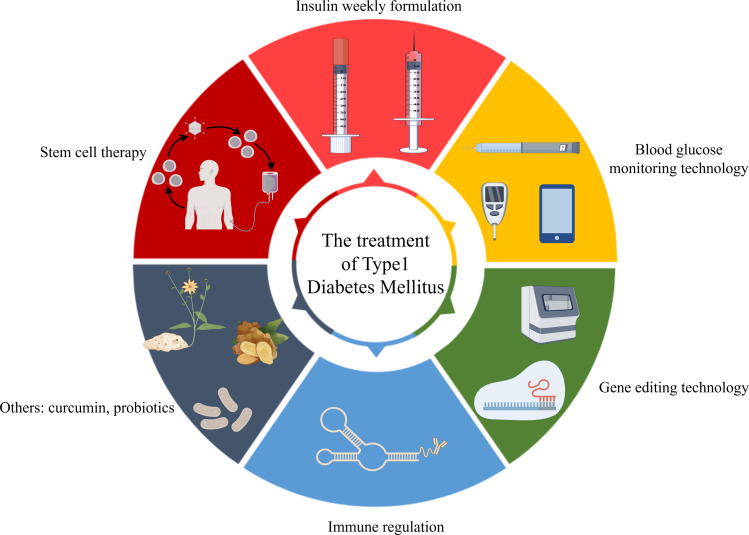

## 1 Introduction

T1DM demonstrates a distinctive epidemiological profile, with incidence predominating in pediatric and adolescent populations, though it may manifest across the lifespan. The escalating global prevalence of T1DM, coupled with its considerable heterogeneity in clinical presentation, disease progression, and complication rates, necessitates a critical reevaluation of contemporary management paradigms. Further impetus for updated guidelines arises from continuous innovations in diabetes technologies and therapeutic agents. This review systematically synthesizes current evidence and evolving strategic approaches in T1DM management, with a dedicated focus on their integration within China’s specific socioeconomic context and healthcare delivery framework.

## 2 Methodology

This narrative review examines recent advancements in type 1 diabetes treatment, including progress, limitations, and future directions of therapeutic technologies. The review adheres to the SANRA (1) guidelines to ensure methodological rigor. A systematic search was conducted from January 2016 to September 2025 across four databases: PubMed, Web of Science, Embase, and Cochrane Library.

The search strategy combined Medical Subject Headings (MeSH) and free-text terms such as “type 1 diabetes”, “stem cell therapy”, “gene editing”, and “immunosuppression”. Inclusion criteria were: original, peer-reviewed English-language research on novel T1DM treatments with high-quality study designs. Exclusion criteria included: non-therapeutic studies, case reports, and methodologically weak research. The systematic screening and deduplication process ensured a high-quality, relevant literature base for subsequent analysis.

## 3 Overview of T1DM

### 3.1 T1DM epidemiology

T1DM is an autoimmune disease characterized by the destruction of pancreatic β-cells, leading to absolute insulin deficiency and a predisposition to ketoacidosis (2).

Globally, the prevalence of T1DM is increasing. The 2021 Global Burden of Disease Study reported 9.6 million individuals with T1DM worldwide, with over 530,000 new cases (3). The International Diabetes Federation (IDF) noted a significant increase in young patients (under 20), from 1.52 million to 1.80 million between 2022 and 2024. The number of T1DM patients aged 65 and older has also nearly tripled over the past three decades (4).

There are significant regional differences in T1DM incidence (5). Nordic countries report the highest rates, with Finland at 62.5 per 100,000 person-years, while Asia has the lowest. However, due to its large population, Asia accounts for approximately one-third of the global T1DM prevalence (6). In China, the incidence has increased notably, nearly quadrupling among children under 15 over the past two decades. Despite a low incidence rate (7), China ranks fourth globally in the number of children and adolescents with T1DM (8). A study published in JAMA (9) revealed that the prevalence of T1DM among adolescents and adults in the United States from 2019 to 2022 was 3.5‰ and 5.3‰, respectively. These figures provide important references for global T1DM epidemiological research.

Over the past 30 years, both the prevalence and incidence of T1DM have continued to rise. In-depth study of the epidemiological characteristics of T1DM is essential for understanding its pathogenesis, developing targeted prevention and treatment strategies, improving patient outcomes, reducing the societal healthcare burden, and contributing significantly to global public health efforts.

### 3.2 Staging and diagnostic criteria for T1DM

**Figure 1:**
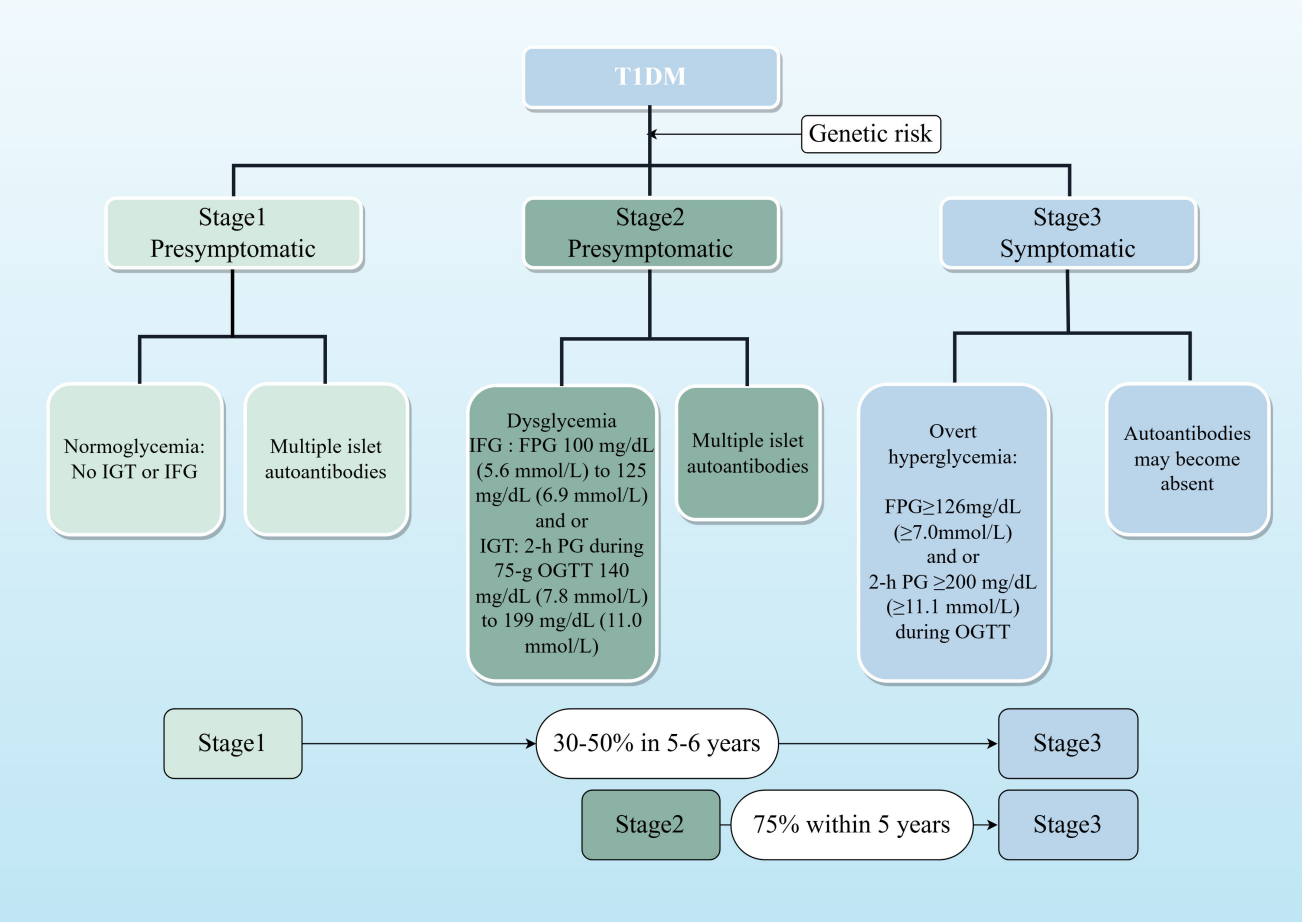
Staged model of T1DM progression. This flowchart illustrates the natural history of T1D, from initial autoimmunity triggered in genetically susceptible individuals to eventual clinical diagnosis. Stages 1 and 2 represent the presymptomatic period, during which intervention may delay progression. The percentages indicate the risk of advancing to the next stage within the specified timeframe, based on ADA 2024 guidelines.

T1DM staging, as defined by the 2024 American Diabetes Association (ADA) guidelines, classifies the disease into three stages based on the presence of autoantibodies and glycemic status (10) (see **Figure 1**). Stages 1 and 2 are latent phases, while Stage 3 marks the onset of clinical T1DM. Progression rates are significant, with 35%–50% of Stage 1 patients advancing to clinical T1DM within 5–6 years, and 75% of Stage 2 patients progressing within 5 years (10). It is important to note that these guidelines are primarily based on data from North American and European cohorts, necessitating adaptation for the Chinese population.

T1DM is a heterogeneous disease, and classifying patients into distinct endotypes can facilitate precision medicine. Two main endotypes, T1DE1 and T1DE2, have been proposed based on differences in immune-cell infiltration, proinsulin processing, and β-cell destruction (11).

T1DE1 is a more aggressive form typically diagnosed in early childhood. It is characterized by rapid and extensive β-cell destruction, abundant CD8^+^T and CD20^+^B lymphocytes, and a high circulating proinsulin-to-C-peptide ratio due to defective proinsulin processing (11) (see **Table 1**).

**Table 1:** Comparison of characteristics of T1DM endotypes.

Feature dimension	T1DM endotype 1 (T1DE1)	T1DM endotype 2 (T1DE2)
Age at Diagnosis	≤7 years (predominantly)	≥13 years (predominantly)
Immune Infiltration	Aggressive insulitis with abundant CD8^+^ T cells and CD20^+^ B cells	Fewer infiltrating CD8^+^ T cells and CD20^+^ B cells
Beta Cell Destruction	Extensive and early	Relatively preserved, many residual insulin-containing islets
Proinsulin Processing	Abnormal	Normal
Key Biomarker	Significantly elevated circulating proinsulin-to-C-peptide ratio	Lower circulating proinsulin-to-C-peptide ratio
Rate of Progression	Typically faster	Typically slower

T1DE2 is a less aggressive form diagnosed in adolescence or adulthood. It is characterized by preserved β-cell mass, minimal insulitis with scarce CD8^+^T and CD20^+^B cells, and a lower proinsulin-to-C-peptide ratio due to largely normal proinsulin processing (11) (see **Table 1**).

**Figure 2:**
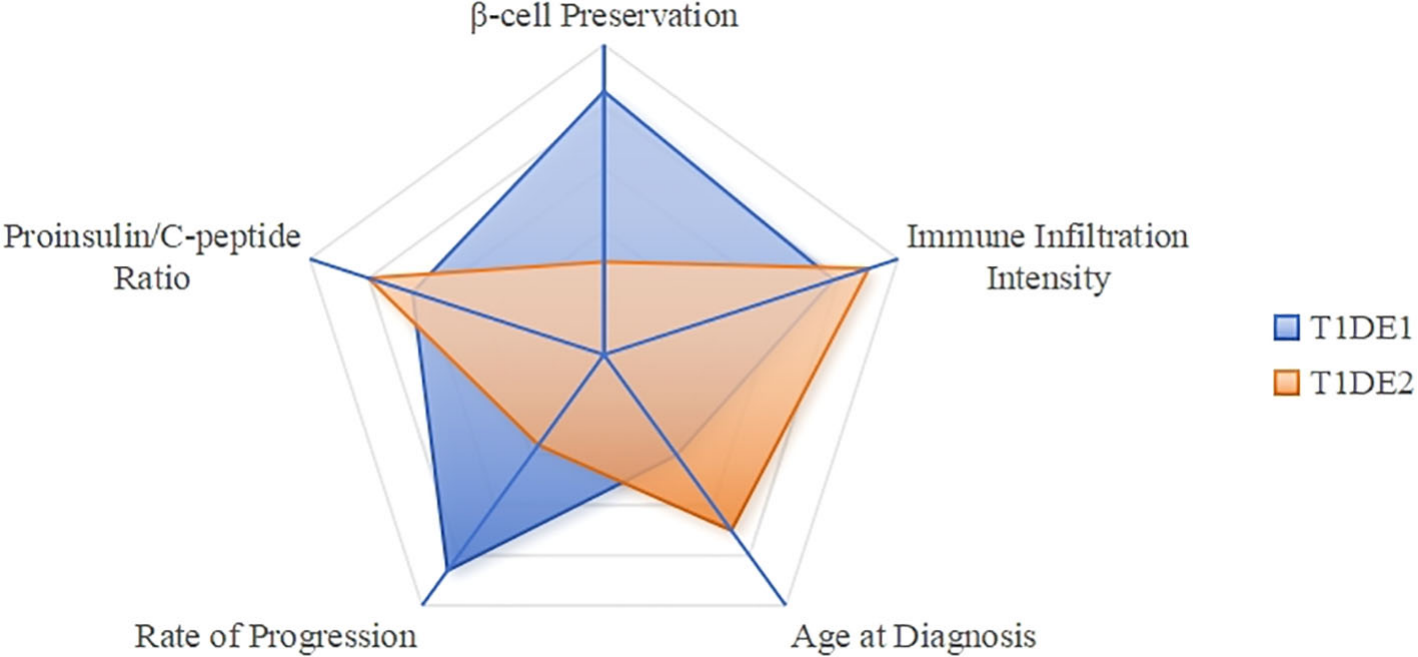
T1DM endotype characteristics comparison radar chart. T1DE1 (Blue): Scores high on “Age at Diagnosis” (low), “Immune Infiltration Intensity” (high), and “Proinsulin/C-peptide Ratio” (high), forming a sharp profile that indicates its aggressive and early-onset characteristics. T1DE2 (Aurantia): Scores high on “Beta Cell Preservation” (high), while scoring lower on other metrics, resulting in a flatter profile that reflects its slow and mild nature.

The proinsulin-to-C-peptide ratio can help differentiate between T1DE1 and T1DE2, particularly in children aged 8–12 years, where both endotypes may coexist. While T1DE1 is more common in children diagnosed before age 7, T1DE2 becomes dominant after age 7 and accounts for most cases in individuals over 13 (11) (see **Table 1**, **Figure 2**).

The endotype concept remains an area of active research. Future studies are needed to validate these subtypes in larger, ethnically diverse cohorts and to identify biomarkers that predict therapeutic responses.

## 4 Latest advances in T1DM treatment

Significant advancements in T1DM management include innovations in insulin formulations, glucose monitoring, automated insulin delivery systems, stem cell therapy, immunomodulation, and gene editing.

### 4.1 Advances in insulin therapy

Recent developments in insulin therapy focus on ultra-long-acting and weekly formulations designed to improve glycemic stability and simplify dosing regimens (12–14).

#### 4.1.1 Insulin degludec

With a half-life of 25 hours, this ultra-long-acting insulin provides a stable basal insulin supply, achieving steady-state plasma concentration in 2–3 days (15). Approved in China for adults with T2DM and in the US and Europe for T1DM patients aged ≥1 year, studies show it offers similar glycemic control and hypoglycemia risk to insulin glargine U300 but at a lower daily dose (16).

#### 4.1.2 Insulin glargine U300

A concentrated form of insulin glargine U100 with a half-life of 19 hours and a duration of action of 36 hours. Approved in China for T2DM and in the US and Europe for T1DM patients aged ≥6 years (17).

#### 4.1.3 Dual insulin analogs

Currently marketed insulin degludec and insulin aspart dual formulations provide a continuous and stable basal insulin supply through the degludec component. The FDA has approved its use for T1DM in patients ≥1 year old; the EMA for those ≥2 years old; in China, it is currently only approved for treating adults with T2DM.

#### 4.1.4 Weekly insulin formulations

Weekly insulin formulations aim to reduce the burden of daily injections, though their use in T1DM is still under evaluation due to a higher risk of hypoglycemia.

**(1) Insulin Icodec**

This weekly insulin binds reversibly to albumin, extending its half-life to 196 hours. While effective at lowering HbA1c, ONWARDS trial data (18, 19) in T1DM patients showed a significantly higher incidence of hypoglycemia compared to daily insulin degludec. It is not currently approved for T1DM in China due to these safety concerns.

The EMA product information for Icodec clearly states its usage and dosage: for T1DM, it must be combined with a short-acting insulin to meet prandial insulin needs; for T2DM patients, it can be used alone or in combination with other insulins.

In short, T1DM patients should use it more cautiously due to safety concerns, and clinical judgment is required.

**(2) Weekly insulin Efsitora alfa (basal insulin Fc)**

A fusion protein that extends its half-life to 17 days. A Phase II trial (20) in T1DM patients demonstrated that weekly dosing was non-inferior to daily insulin degludec for glycemic control, but close monitoring for hypoglycemia was required, particularly at the start of treatment (21).

The pharmacokinetic parameters and applicable populations of several long-acting insulins are detailed in **Table 2**.

**Table 2:** Comparison of different long-acting insulins for T1DM.

Insulin preparation	Onset (h)	Half-life (h)	Duration (h)	Approval status&age (years)		Dose range (U)
Degludec	1.0	25	42	Not approved in China	FDA/EMA: ≥1 y	3 mL: 300
Glargine U300	6.0	19	36	China: ≥6 y, adults	FDA/EMA: ≥6 y	1 mL: 300
Degludec/Aspart	1–2	25	24–36	Not approved in China	FDA: ≥1 y, EMA: ≥2 y	3 mL: 300
Icodec	–	192	>168	Not approved in China	FDA: adults only	1 mL: 700
Efsitora alfa	–	408	–	Not approved		–

### 4.2 Innovations in blood glucose monitoring technology

Smart insulin pens and continuous glucose monitoring (CGM) systems are a cornerstone of modern diabetes management. These technologies work together to significantly improve the accuracy and convenience of T1DM treatment by moving beyond manual tracking and fingerstick blood sugar measurements (22) (23).

Connected insulin pens, like the NovoPen^®^ 6, automatically log dose data, including the time and amount of each injection. When used with a CGM, which provides a continuous stream of real-time glucose data, these devices offer a comprehensive view of a patient’s glycemic patterns. This integrated data allows both patients and clinicians to identify trends, optimize insulin timing, and improve overall adherence to treatment plans. A clinical study showed that using a connected pen reduced missed injections by 43% and increased Time in Range (TIR) by 8.5%, while decreasing both Time Above Range (TAR) and Time Below Range (TBR) (24).

Beyond simple data logging, smart insulin pens can provide dose suggestions based on a patient’s individual settings, helping to prevent dosing errors that can lead to hyperglycemia or hypoglycemia. They can also track insulin on board (IOB), or active insulin, to prevent stacking insulin doses and distinguish between priming and therapeutic injections. This functionality provides a more accurate picture of a patient’s insulin needs and delivery patterns, which is essential for adjusting basal insulin doses. The development of these devices is focused on further integration with CGM data to provide more proactive, personalized alerts and reduce the burden of daily management (22).

### 4.3 Closed-loop systems and artificial intelligence

CGM, Automated Insulin Delivery (AID) systems, fully closed-loop technology, and AI-powered decision support systems represent the future direction of T1DM management, capable of significantly improving the quality of glycemic control and patient quality of life. However, their widespread adoption remains limited by numerous practical factors such as cost, technical reliability, regulatory frameworks, data security, and healthcare accessibility. Addressing these challenges in the future requires interdisciplinary collaboration, particularly focused on reducing costs and improving system interoperability, while simultaneously accumulating more real-world evidence and developing corresponding regulatory guidelines and training systems to ensure all T1DM patients can benefit equitably from these innovative technologies.

#### 4.3.1 Integration of real-time continuous glucose monitoring and automated insulin delivery systems

Integration of Continuous Glucose Monitoring (CGM) and Automated Insulin Delivery (AID) systems represents a major leap forward in T1DM management. These technologies combine to create a hybrid closed-loop system that automates basal insulin delivery based on real-time glucose data, significantly improving glycemic control and reducing the burden of manual management (25, 26).

The ADA (American Diabetes Association) (27) Standards of Medical Care in Diabetes recommend AID systems for both adolescents and adults with T1DM. Clinical studies, such as the ADAPT study (28), have provided strong evidence for their effectiveness. This study demonstrated that an AID system not only achieved a 1.4% reduction in HbA1c but also a 26.7% increase in Time in Range (TIR) over six months compared to standard multiple daily injections (MDI) with intermittent CGM. These systems automate background insulin adjustments, but still require users to manually input mealtime boluses (29).

However, the widespread adoption of AID systems faces several challenges. The high cost of the devices and consumables, along with limited insurance reimbursement, creates a significant financial burden. Additionally, compatibility issues between different brands of CGM and insulin pumps hinder seamless integration. The complexity of these systems also requires extensive training for both patients and healthcare professionals to ensure safe and effective use.(see **Table 3**).

**Table 3:** Comparison of different blood glucose management devices.

Devices	Features	Limitations
CGM + AID integration	Increases TIR, lowers HbA1c, reduces hypoglycemia	High cost, limited insurance coverage, technology-compatibility issues, user-operation complexity
Fully closed-loop AID system	Automates glucose management, lessens daily patient burden	Algorithms need optimization for complex scenarios; long-term device reliability unproven; DIY systems carry safety & regulatory risks
AI insulin decision-support system	Offers personalized dose advice, improves control in special situations (high-fat meals, exercise)	Data privacy & security concerns, “black-box” algorithms erode trust, lack of large-scale long-term RCT evidence, difficult to integrate into existing clinical workflows

#### 4.3.2 Fully closed-loop automated insulin delivery systems

Fully closed-loop automated insulin delivery (AID) systems, often referred to as an “artificial pancreas,” are designed to fully automate T1DM management by linking a continuous glucose monitor (CGM) to an insulin pump. Unlike hybrid systems, these advanced algorithms aim to eliminate the need for manual insulin boluses at mealtimes, providing a truly hands-off approach to blood glucose control. Studies have shown these systems can lower average HbA1c to around 6.9%, a level below the standard target for many adults with T1DM (30).

Despite their promise, artificial pancreas systems face several challenges. The algorithms have limited ability to adapt to physiological events like exercise or illness, and their long-term reliability and accuracy still require improvement (31). There are also concerns about the stability of implanted sensors and the precision of insulin pumps over time. Additionally, patient-developed “Do-It-Yourself” (DIY) closed-loop systems—while demonstrating impressive results, such as increasing Time in Range (TIR) to 82.4% and reducing HbA1c to 6.2%—lack regulatory approval and long-term safety data, introducing potential risks (32). Further research and regulatory oversight are necessary to validate these systems for broader clinical use.

#### 4.3.3 Artificial intelligence

Artificial intelligence (AI), specifically reinforcement learning (RL), is emerging as a powerful tool for personalized insulin dose adjustment in T1DM. The iBolusV2 mobile app, for example, uses RL algorithms to provide individualized insulin recommendations for complex scenarios like high-fat meals and post-meal exercise. A 16-week study (33) demonstrated that this system could improve glycemic control in these specific situations, showing the potential for AI to enhance T1DM management beyond traditional methods.

However, the path to widespread adoption for AI in T1DM care is challenging. Key hurdles include (33):

Data Privacy: AI systems require access to large amounts of sensitive patient data, making privacy and security a primary concern.

Trust and Transparency: The “black box” nature of complex AI algorithms can make their decision-making process difficult to understand, which can erode trust among both clinicians and patients.

Validation: Most existing research is based on small-scale studies. More extensive, long-term randomized controlled trials are needed to confirm the safety and effectiveness of these systems.

Clinical Integration: Seamlessly incorporating AI-based decision support into existing clinical workflows presents a significant logistical challenge.

#### 4.3.4 Limitations of diabetes management techniques

Advancements in diabetes technology face significant limitations that hinder their widespread adoption. While devices like continuous glucose monitoring (CGM) and automated insulin delivery (AID) systems are effective, their real-world application is constrained by several factors, including global disparities in access, high costs, and challenges with patient adherence (see **Table 3**).

Despite advancements in diabetes technology, significant limitations impede equitable global implementation. A primary concern is the stark disparity in access, wherein developed nations and urban centers possess substantially greater resources compared to developing regions and rural areas, a gap exacerbated by infrastructural and economic constraints. Furthermore, the prohibitive costs of devices, consumables, and maintenance, often inadequately covered by insurance, pose a major financial barrier. Finally, patient adherence remains challenging due to the complexity of systems, a need for continuous education, and insufficient technical support. Overcoming these barriers necessitates the development of cost-effective and user-friendly technologies, expanded healthcare access, and robust support systems to ensure broad and equitable patient benefit (see **Table 3**).

### 4.4 New advances in stem cell therapy

Recent advances in stem cell therapy for T1DM, particularly in the United States, focus on using stem cell-derived, fully differentiated, insulin-producing islet cells to replace destroyed pancreatic beta cells. These therapies aim to restore natural insulin production and eliminate the need for exogenous insulin.

#### 4.4.1 Zimiselcel (VX-880)

Vertex Pharmaceuticals’ Zimiselcel (VX-880) is a promising allogeneic islet cell therapy. In a Phase 1/2 clinical trial, all patients receiving the full dose showed successful islet cell engraftment and restored endogenous C-peptide secretion, which led to the disappearance of severe hypoglycemic events and significant reductions in insulin use. A notable result was that 7 out of 10 patients achieved complete insulin independence by day 180, and 10 of 12 patients were insulin-independent at day 365, demonstrating the potential for long-term efficacy (34).

Stem cell-based therapies, while holding considerable therapeutic promise, are confronted with substantial safety concerns and limitations that must be addressed before widespread clinical application. A major impediment is immune-mediated rejection of allogeneic grafts, which necessitates long-term systemic immunosuppression. This regimen heightens the risk of opportunistic infections, nephrotoxicity, and malignancy (34). Another critical safety consideration is the potential for tumorigenicity, including teratoma formation, arising from residual undifferentiated pluripotent stem cells within the transplant—a risk that, though not yet evident in clinical trials, mandates rigorous long-term monitoring. Further practical constraints include the high complexity and cost associated with manufacturing and delivering these treatments, significantly limiting their scalability and accessibility. Moreover, current clinical evidence remains constrained by studies with small cohorts and limited follow-up duration, underscoring the necessity for extended observational data to thoroughly evaluate the long-term durability and safety profile of these interventions.

#### 4.4.2 Encapsulated stem cell-derived islet cell therapy

This approach involves encapsulation within an immune-protective device to achieve physical immune isolation, aiming to maintain cell function without the need for long-term immunosuppression.

An open-label Phase 1/2 trial (NCT03163511) (35) enrolled 10 C-peptide negative T1DM patients who were implanted with devices loaded with a high dose of islet cells. At 6 months post-operation, 4 patients showed C-peptide secretion, with 3 maintaining C-peptide ≥0.1 nmol/L for one year. Insulin use significantly decreased, TIR increased from 55% to 85%, and daily insulin dose was reduced by 44%. No transplant rejection or serious adverse events were observed.

Although encapsulated cell therapy aims to avoid systemic immunosuppression through physical isolation, it still faces several potential long-term risks (35). The encapsulation device may gradually lose function due to material biodegradation, fibrotic encapsulation, or immune cell infiltration. Even under encapsulation, chronic inflammatory responses in the local microenvironment may persist, affecting the viability and long-term functional stability of the implanted cells (35). Furthermore, if the integrity of the encapsulation structure is compromised, allowing abnormal migration or proliferation of internal cells, a theoretical risk of tumorigenicity remains. Crucially, the concept of being “free from immunosuppression” in this strategy has not yet been fully validated in large-scale clinical trials; its long-term safety and immune evasion effects still require further rigorous evaluation.

#### 4.4.3 CiPSC: chemically reprogrammed induced pluripotent stem cell therapy

The groundbreaking study (36) by Professor Shen et al. in September 2024 represents a significant advance in personalized T1DM treatment using chemically induced pluripotent stem cells (CiPSCs). The research successfully used chemical reprogramming to convert a T1DM patient’s adipose tissue into functional islet cells, which were then transplanted back into the same patient. This approach avoids the need for external gene integration, enhancing its theoretical safety.

The clinical data from this study (36) were highly promising. Within 75 days post-transplant, the patient achieved complete insulin independence, which was maintained for a full year of follow-up. Glycemic control demonstrated marked enhancement following intervention: Time in Range (TIR) exceeded 98% within four months, and HbA1c declined from a baseline of 7.57% to a non-diabetic level below 5.7%. This study substantiates the feasibility and efficacy of transplanting CiPSC-derived islet cells, particularly via a novel subfascial technique that facilitates easier management and monitoring, thereby offering a promising direction for future islet transplantation protocols. Nevertheless, the clinical translation of this autologous cell therapy faces several pivotal challenges (37). Key among these is safety, wherein despite the avoidance of genetic modification, persistent concerns regarding tumorigenicity—potentially arising from residual pluripotent cells or epigenetic anomalies—necessitate extended longitudinal surveillance. Additionally, the underlying autoimmune pathology may lead to recurrence of beta-cell destruction unless concomitant immune modulation is achieved. Finally, scalability remains a major impediment, as the personalized nature of the treatment entails a complex, costly, and time-intensive manufacturing process, hindering standardized production and broad implementation.

In summary, the three stem-cell platforms each possess distinct advantages and limitations, with significant differences in their efficacy and risk (see **Table 4**). **Table 4** provides a direct comparison of these technologies and can serve as a reference for future clinical translation and trial design.

**Table 4:** Comparison of several stem cell therapies.

Product name	Product type	Source	Indication	Immune suppression	Allogeneic/ autologous	Sponsor	Study phase	Start date	Clinical references
Zimislecel	pancreatic islet cells	HESC	T1DM	Yes	Allogeneic	Vertex Pharmaceuticals	I-II	2021	Reichman T W et al. (35)
VX-264	pancreatic islet cells (VX-880, encapsulated)	HESC	T1DM	No	Allogeneic	Vertex Pharmaceuticals	I/II	2023	-
CiPSC islets	islet-like cells	HiPSC	T1DM	Yes	Autologous	Tianjin First Center Hospital	I	2023	Wang et al. (47)

#### 4.4.4 Mechanisms of stem cell therapy

**Figure 3:**
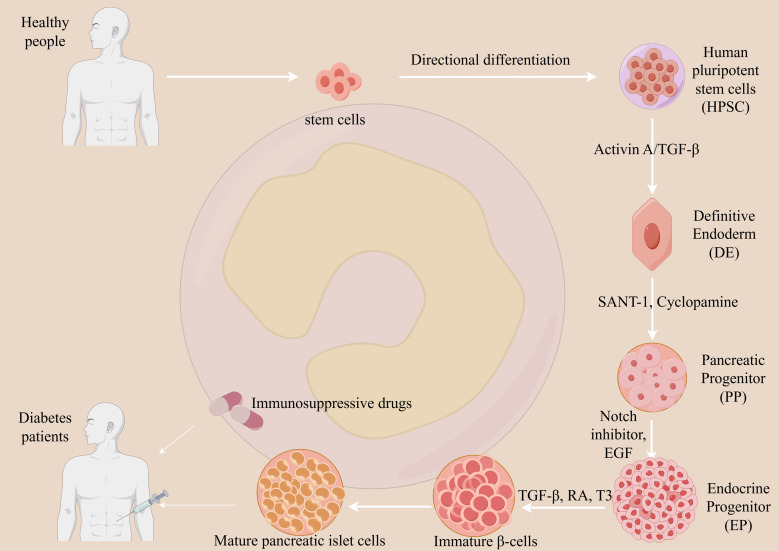
Mechanism of stem cell therapy for T1DM. Stem cell therapy aims to restore endogenous insulin production in T1DM through cell replacement. Pluripotent stem cells (PSCs) are differentiated *in vitro* into glucose-responsive, insulin-producing cells via a multi-stage process mimicking pancreatic development. Key steps involve directing PSCs toward definitive endoderm and pancreatic progenitors using signaling molecules such as Activin A and retinoic acid, followed by endocrine induction with small molecule inhibitors. The resulting islet-like cells are transplanted into the patient. However, due to the autoimmune etiology of T1DM, the transplanted cells remain vulnerable to immune destruction. Therefore, lifelong immunosuppression is typically required to protect the graft from rejection and ensure its survival and function. This approach seeks to re-establish physiological insulin secretion and improve metabolic control.

Stem cell therapy for T1DM, based on the principle of cell replacement, aims to provide a functional cure by replacing destroyed pancreatic β-cells. This process involves the *in vitro* differentiation of pluripotent stem cells (PSCs) into glucose-responsive, insulin-producing cells (see **Figure 3**).

*In Vitro* Differentiation: PSCs, such as embryonic stem cells (ESCs) or induced pluripotent stem cells (iPSCs), are carefully guided through a series of stages to mimic embryonic pancreatic development. This involves using specific growth factors to produce mature, functional islet-like clusters (34).

*In Vivo* Engraftment: The differentiated cells are then transplanted into the patient, typically via infusion into the portal vein or implantation within a protective device. Once engrafted, these new cells restore a natural, self-regulating feedback loop by sensing glucose levels and secreting insulin as needed (35).

This “cell factory replacement” strategy has the potential to eliminate the need for insulin injections and reduce diabetes-related complications. However, two primary challenges remain: ensuring the purity of the differentiated cells to prevent tumor formation and, for allogeneic transplants, overcoming immune rejection.

#### 4.4.5 Limitations of stem cell therapy

Stem cell-derived islet cell therapy represents a promising treatment avenue for T1DM; however, its long-term safety profile and widespread clinical application face considerable challenges. Chief among these concerns is the risk of tumorigenicity, particularly teratoma formation originating from residual undifferentiated pluripotent stem cells, which necessitates stringent quality control during manufacturing and mandates long-term post-transplant imaging surveillance. Immune rejection also remains a critical barrier: both allogeneic and autologous transplants are susceptible to chronic rejection and recurrent autoimmunity, even with the use of immunosuppressive regimens or encapsulation technologies. Furthermore, the evidence base is currently constrained by a scarcity of large-scale, long-term studies, with most clinical trials being short-term and involving limited cohorts, thereby impeding a comprehensive assessment of long-term efficacy and safety. Large prospective trials are urgently needed to establish a reliable benefit-risk profile. Additionally, the therapy’s high costs and limited scalability—stemming from a complex, labor-intensive production process—pose substantial obstacles to standardization and broad accessibility. Future research should prioritize the refinement of differentiation protocols, the development of effective immune-evasion strategies, enhanced safety monitoring platforms, and more efficient manufacturing processes to facilitate clinical translation and equitable adoption.

### 4.5 New advances in immunomodulatory therapy

Teplizumab (Tzield) is a groundbreaking immunomodulatory drug that targets the underlying autoimmune process of T1DM. It works by binding to CD3 on the surface of T cells, which are responsible for destroying the insulin-producing pancreatic β-cells (38) (see **Table 5**). This action helps to modulate the immune response, preserving β-cell function and delaying the progression to clinical T1DM.

**Table 5:** Comparison of Teplizumab, TrialNet oral insulin, and Hypoimmunogenic islet cell therapy.

Name	Teplizumab	Trialnet oral insulin	Hypoimmunogenic islet cell therapy
Mechanism of Action	Anti-CD3 monoclonal antibody; modulates T-cells to delay autoimmune destruction of β-cells.	Oral mucosal immune tolerance; induces antigen-specific immune regulation to insulin.	Gene-edited allogeneic islet cells with reduced immunogenicity (e.g., MHC I/II knockout, CD47 overexpression).
Target Population	Stage 2 T1DM (autoantibody positive, dysglycemia) patients ≥8 years old.	High-risk individuals (autoantibody positive) for preventing progression to clinical T1DM.	Patients with established T1DM (insulin-dependent) aiming to restore endogenous insulin production.
Primary Goal	Delay progression from Stage 2 to Stage 3 (clinical) T1DM.	Delay or prevent onset of clinical T1DM in high-risk individuals.	Replace destroyed β-cells; achieve insulin independence without long-term immunosuppression.
Route of Administration	Intravenous infusion (14-day course).	Oral administration.	Intraportal infusion or implantation via encapsulation device.
Immunosuppression	Not required.	Not required.	Not required.
Key Efficacy Data	TN-10 study: delayed median T1DM diagnosis by ~25 months vs placebo.	TN-07 trial: delayed onset in subgroups (high IA-2A; HLA-DR4/DQ8+).	Preclinical (primate): Insulin independence for 6 months. Early human trials (UP421): C-peptide detection post-transplant.
Major Advantages	First disease-modifying therapy to delay clinical T1DM; non-invasive administration.	Non-invasive; targets specific immune tolerance; potential for prevention.	Potential for restoring physiological insulin secretion; “off-the-shelf” universal donor concept.
Major Limitation/Challenge	Does not prevent or cure T1DM; only delays onset; limited to Stage 2 patients; cost.	Modest effect size; efficacy primarily in specific subgroups.	Theoretical tumorigenicity risk (teratoma); potential need for safety switches; immune evasion durability; complex manufacturing; high cost.
Stage of Development	Approved (FDA, 2022) for Stage 2 T1DM.	Clinical Trials (Phase III completed, subgroup analysis).	Early Clinical Research (Phase I/II trials ongoing, e.g., UP421 NCT06239636).
Reference	(38) (39),	(41)	(42) (43),

This therapy is a significant advance because it is the first drug approved by the FDA to delay the onset of Stage 3 T1DM in high-risk individuals (those aged 8 years and older with Stage 2 T1DM) (27). A single 14-day course of Teplizumab has been shown (39) to delay the median time to diagnosis of insulin-dependent diabetes by approximately two years. This delay is particularly important for children, who face significant challenges with lifelong disease management (40).

Another notable immunomodulatory approach is the use of oral insulin, which aims to induce immune tolerance. While a trial (41) showed that oral insulin did not significantly delay T1DM in the overall high-risk population, it did show a significant effect in a specific subgroup with high levels of IA-2 autoantibodies. These complementary approaches, both disease-modifying therapies, represent a new paradigm of proactive, rather than reactive, T1DM care.

In summary, Teplizumab has become the first approved disease-modifying therapy for T1DM by virtue of its targeted immunomodulation that significantly delays clinical onset, whereas oral insulin demonstrates antigen-specific tolerogenic potential in defined high-immunologic-risk subgroups. These complementary approaches expand the preventive/delay armamentarium across the T1DM staging spectrum (see **Table 5**).

### 4.6 Potential of gene editing technology

#### 4.6.1 Hypoimmunogenic islet cell therapy

Gene editing is a cutting-edge approach to T1DM treatment that offers a potential solution to immune rejection, a major hurdle for cell replacement therapies. Hypoimmunogenic islet cell therapy uses gene editing, like CRISPR, to modify donor cells. The process involves two key steps (42):

Immune Evasion: Genes responsible for immune recognition, such as MHC I and MHC II, are knocked down or silenced. This makes the transplanted cells “invisible” to the host’s T cells, preventing an adaptive immune attack.

Immune Tolerance: Genes are added to overexpress molecules like CD47, which sends a signal to the innate immune system (e.g., macrophages), preventing cell destruction.

Sana Biotechnology has shown (42) promising preclinical data in non-human primates using this approach. Their engineered pseudo-islets were able to achieve insulin independence for six months without immunosuppression. They also demonstrated a built-in “safety switch” where the grafts could be eliminated with an anti-CD47 antibody, addressing a key safety concern. The results of the first human trial are also very promising, with the transplanted cells showing survival, function, and immune evasion in the absence of immunosuppression.

#### 4.6.2 UP421

UP421 is a groundbreaking allogeneic islet cell therapy that uses gene editing to create Hypoimmune platform (HIP) islet cells (43). The goal of UP421 is to allow for islet transplantation to treat T1DM without the need for long-term immunosuppression.

This therapy uses the CRISPR-Cas12b system to knock out the genes for HLA Class I (B2M) and Class II (CIITA) molecules, which are the primary targets of the immune system. Additionally, the cells are engineered to overexpress the CD47 protein, which sends an innate immune “don’t eat me” signal. The initial clinical trial results are promising, showing that the transplanted cells survived, produced insulin (indicated by C-peptide levels), and successfully evaded immune detection for at least 12 weeks (43).

While these early findings are a significant step forward, they are preliminary and need to be validated with longer-term data. The goal of this research is to move toward a scalable, off-the-shelf solution for T1DM.

### 4.7 Gamma-aminobutyric acid (GABAergic system) as a novel target for T1DM treatment

The GABAergic system is a promising, novel target for T1DM treatment due to its role in β-cell survival, regeneration, and immune regulation (44). By activating GABA receptors on β-cells, researchers believe they can promote insulin release, increase β-cell proliferation, and prevent apoptosis. Additionally, GABA can reduce inflammatory cytokine production and inhibit T-cell proliferation, which may protect β-cells from autoimmune attack (45).

While this research is still experimental, early clinical and preclinical studies have provided promising results. For example, a study on newly diagnosed children with T1DM showed that oral GABA, alone or with GAD, was well-tolerated and met a secondary outcome of reduced serum glucagon. Another study combining GABA with a DPP-4 inhibitor and a PPI showed improved glycemic control and reduced insulin requirements in some patients (45). However, a separate Phase I/II trial using a controlled-release GABA formulation did not show improvement in endogenous insulin production. This highlights that while the potential is great, more research is needed to determine the right formulation, dosage, and patient population for effective treatment.

### 4.8 Phytomedicine: anti-inflammatory effects and therapeutic potential of curcumin in T1DM

Curcumin, a natural compound from turmeric, holds therapeutic potential for T1DM due to its anti-inflammatory, antioxidant, and immunomodulatory properties (46). It has been shown in preclinical studies to improve β-cell function, inhibit cell death, and reduce immune infiltration into the pancreas (47, 48). This could potentially delay the onset and progression of the disease (49, 50).

The main challenges for curcumin’s clinical use are its poor water solubility and low bioavailability, which limit its absorption and effectiveness in the body (51). However, researchers are developing new delivery systems, such as nano-formulations, to overcome these limitations and improve its therapeutic potential (52).

### 4.9 Application of probiotics and gut microbiota modulation in T1DM prevention

The use of probiotics and gut microbiota modulation in preventing and treating T1DM is an exciting area of research, based on the link between gut microbiota dysbiosis and T1DM pathogenesis (53). The core idea is that an imbalance in gut bacteria may contribute to the autoimmune attack on pancreatic β-cells by affecting gut permeability and immune regulation (54).

Emerging research suggests that gut microbiota modulation may offer a promising avenue for intervening in T1DM (55, 56). Probiotic supplementation has been shown in animal models, such as NOD mice, to mitigate diabetes development through mechanisms involving enrichment of beneficial bacteria and enhanced intestinal barrier integrity (57). Preliminary clinical studies further indicate that specific probiotics can reduce systemic inflammatory markers and may attenuate β-cell autoimmunity in high-risk individuals (58). Beyond probiotics, fecal microbiota transplantation (FMT) represents a more invasive yet potentially effective strategy to reconstitute the gut microbial community. Early-phase studies report that FMT may help preserve residual β-cell function in recent-onset T1DM. Nonetheless, this field remains in its nascent stages. Significant challenges include identification of optimal microbial strains, standardization of treatment protocols, and a pressing need for large-scale randomized trials to validate long-term efficacy and safety (53).

### 4.10 Limitations of current treatments

New and emerging T1DM therapies, while promising, face significant challenges in moving from research to widespread clinical use. The primary limitations for each type of therapy are distinct:

#### 4.10.1 Stem cell therapy

Stem cell-based therapies, such as the use of allogeneic (donor) or autologous (patient-derived) cells, face major hurdles related to safety and scalability. The main safety risks are tumorigenicity from undifferentiated cells and immune rejection of the transplanted graft, which can lead to the need for lifelong immunosuppression. From a practical standpoint, the complexity, high cost, and lack of standardization in the manufacturing process severely limit their clinical adoption and accessibility.

#### 4.10.2 Immunomodulatory and gene-editing therapies

Immunomodulatory drugs like Teplizumab can delay disease progression by targeting the immune system but are not a cure and are only effective in a specific population of early-stage T1DM patients (59). The long-term safety of these drugs also needs to be established. Gene-editing approaches, like hypoimmunogenic cells, offer a potential solution to immune rejection, but concerns remain about the safety of the gene-editing process itself, including off-target effects and the potential for these cells to evade immune surveillance if they become malignant.

#### 4.10.3 Other novel therapies

Other promising approaches like GABA-system modulation and curcumin are still in the early stages of research. They show potential for β-cell protection and immune modulation in preclinical studies but lack large-scale human clinical trial data. These therapies also face challenges with bioavailability and may not be effective as a single-agent treatment.

## 5 Prognosis and management of T1DM

Predicting and preventing T1DM is a critical area of research with significant international progress, yet it faces notable hurdles, particularly in China. The core of prediction relies on identifying high-risk individuals before clinical symptoms appear, primarily through islet autoantibody testing combined with genetic and metabolic markers. This allows for a tiered prevention strategy.

### 5.1 Prediction and prevention of T1DM

In recent years, T1DM prediction research has made significant progress internationally. Strategies integrating islet autoantibodies, genetic susceptibility, and metabolic markers have improved the identification of high-risk individuals for T1DM, enabling better early intervention and trial design (11, 60). However, challenges such as high costs, lack of insurance coverage, and uneven distribution of medical resources hinder the widespread implementation of predictive screening, especially in low- and middle-income countries (61). In China, limited accessibility and standardization of autoantibody testing further restrict its use in large-scale screening.

Current prevention strategies for T1DM comprise a three-tiered framework (27). Primary prevention aims to halt the initiation of autoimmunity in genetically susceptible individuals through modulation of environmental triggers such as diet and gut microbiota. Secondary prevention targets individuals who have developed islet autoantibodies but have not yet progressed to clinical diabetes (Stage 1 or Stage 2 T1DM), employing agents such as Teplizumab to delay overt disease onset (39). Tertiary prevention focuses on preserving residual beta-cell function and delaying complications in newly diagnosed patients via intensive insulin therapy and emerging immunotherapies (62, 63).

### 5.2 Prevention and management of long-term complications

Effectively managing T1DM to prevent long-term complications requires a multifaceted approach that goes beyond just insulin. It’s about combining tight glycemic control with comprehensive lifestyle management and regular monitoring of risk factors.

The DCCT (Diabetes Control and Complications Trial) (64) and its long-term follow-up, the EDIC (Epidemiology of Diabetes Interventions and Complications) study, provide the strongest evidence that intensive insulin therapy significantly reduces the risk of long-term complications. These studies (64) also established the concept of a “legacy effect” or “metabolic memory,” which means that periods of good glycemic control early in the disease course provide a long-lasting protective benefit against complications, even if control wanes later. Conversely, early hyperglycemia can lead to lasting vascular damage.

Modern guidelines from organizations like the ADA and EASD emphasize individualized care (65, 66). This includes setting personalized glycemic targets based on factors like age, history of hypoglycemia, and presence of complications (61). For example, while a general HbA1c target might be <7.0% (12), a more flexible target may be set for a patient with hypoglycemia unawareness.

Comprehensive management (66) of T1DM extends beyond glycemic control and incorporates multidisciplinary approaches, including lifestyle interventions, systematic monitoring, and psychosocial support. Medical nutrition therapy (MNT) delivered by registered dietitians, combined with regular physical activity, enhances insulin sensitivity and may reduce exogenous insulin requirements. Furthermore, consistent complications surveillance—such as routine ophthalmologic examinations, foot assessments, and monitoring of blood pressure and lipid profiles—is critical for early detection and intervention. Finally, given the substantial self-management demands of T1DM, structured psychosocial support and patient education are essential components to improve treatment adherence and overall quality of life.

### 5.3 Self-management education

Self-management education is a crucial aspect of T1DM care, as it empowers patients with the knowledge and skills needed to effectively manage their condition. Guidelines from the American Diabetes Association (ADA) (27) emphasize that all people with diabetes should receive this education to improve clinical outcomes, health status, and quality of life.

The education and self-management support for T1DM have traditionally relied on structured tools (12) such as conversation maps—a visual aid designed to facilitate group-based interactive learning—as well as food models for improving carbohydrate estimation and portion control, and health manuals that deliver essential knowledge regarding nutrition, physical activity, and medication. In recent years, digital health (e-Health) interventions (67, 68) have gained increasing prominence. Devices such as smart insulin pens enable automated logging of dosing and timing information, which can be seamlessly shared with clinicians, while mobile applications analyze real-time data from continuous glucose monitoring (CGM) systems to deliver personalized feedback and alerts. These digital tools show promise in enhancing glycemic outcomes and strengthening self-efficacy. Nevertheless, their broader implementation faces challenges including data privacy concerns, a scarcity of long-term efficacy evidence, and the “digital divide”—inequities in technology access and digital literacy that may exacerbate existing health disparities.

## 6 Conclusions

China is making notable progress in diabetes care through integrating technological innovation and public health strategies, yet considerable challenges persist. While advanced technologies such as continuous glucose monitoring (CGM) and automated insulin delivery (AID) systems are being adopted, a significant gap remains in population-specific clinical evidence and precision medicine guidelines tailored to East Asian patients. To address this, conducting high-quality multicenter clinical trials and developing cost-effective, user-friendly technologies are essential—particularly in resource-limited settings. Government-led initiatives, including the “Shanghai Integration Model” and the “Healthy China 2030” campaign, aim to narrow urban–rural health disparities and promote multidisciplinary diabetes management.

Patient self-management capabilities require further strengthening, with digital health platforms—encompassing remote monitoring and AI-assisted clinical decision support—playing an instrumental role in establishing patient-centered, holistic care systems. Programs such as the “Road to Hierarchical Diabetes Management at Primary Care Settings in China” have demonstrated potential in enhancing care quality; however, issues including the digital divide and data security must be systematically addressed.

China is also engaging in pioneering biomedical research, particularly in stem cell therapy and gene editing. Recent breakthroughs in chemically induced pluripotent stem cell (CiPSC)-derived islet cells represent a promising route toward personalized, immunosuppression-free treatments. Nonetheless, these novel therapies remain in early development and require validation through larger clinical trials. To foster innovation and ensure equitable access, China is strengthening participation in global diabetes governance networks, advocating for data sharing, and contributing to internationally harmonized guideline development.

## Glossary

AI: Artificial Intelligence
ADA: American Diabetes Association
AID: Automated Insulin Delivery
AHCL: Advanced Hybrid Closed-Loop
BIF: Basal Insulin Fc
CDE: Center for Drug Evaluation
CiPSCs: Chemically induced pluripotent stem cells
CGM: Continuous Glucose Monitoring
DCCT: Diabetes Control and Complications Trial
ESCs: Embryonic stem cells
EDIC: Epidemiology of Diabetes Interventions and Complications
EGF: Epidermal Growth Factor
EMA: European Medicines Agency
FBG: Fasting blood glucose
FDA: Food and Drug Administration
HESC: Human embryonic stem cell
HCL: Hybrid Closed-Loop
HIP: Hypoimmunogenic
HIPSc: Hypoimmune induced pluripotent stem cells
IST: Investigator-sponsored trial
IA-2A: Islet antigen-2 autoantibodies
ISCGM: Intermittently Scanned Continuous Glucose Monitoring
IDF: International Diabetes Federation
MNT: Medical nutrition therapy
MDI: Multiple daily injection
OGTT: Oral glucose tolerance test
PSCs: Pluripotent stem cells
RA: Retinoic acid
RL: Reinforcement Learning
TIR: Time in Range
T1DM: Type 1 diabetes mellitus
T2DM: Type 2 diabetes mellitus
T1DE1: Type 1 diabetes mellitus endotype 1
T1DE2: Type 1 diabetes mellitus endotype 2
